# Do they really boil their drinking water? a descriptive study in a rural district of the Lao people’s democratic republic

**DOI:** 10.1186/s41182-024-00626-z

**Published:** 2024-09-18

**Authors:** Sae Kawamoto, Daisuke Nonaka, Nouhak Inthavong

**Affiliations:** 1 Graduate School of Health Sciences, University of the Ryukyus, Okinawa, Japan; 2 Lao Tropical and Public Health Institute, Ministry of Health, Vientiane, Lao PDR

**Keywords:** Water treatment, Health behavior, Self-report, Demographic and Health Survey, Laos

## Abstract

**Background:**

For safe drinking water, household water treatments (HWT) is important to reduce the risk of diarrhea in low-and-middle countries including Lao People’s Democratic Republic (Lao PDR). However, the measurement of HWT relies chiefly on self-report in most nationwide surveys. Thus, the validity of self-reported measurement is of concern. The objective of this study was to determine the proportion of households with the presence of boiled water among households that report boiling practices in a rural area of the Lao PDR.

**Methods:**

This study was conducted with randomly selected 108 households in the four villages in the catchment area of the two health centers, in Xepon district of the Savannakhet province, between September and October 2023. The inclusion criterion of the households was the households that report boiling as HWT. Surveyors conducted interviews with an adult household member and observations on boiled water through household visits, using a questionnaire. Descriptive statistics were conducted to summarize the collected information using the frequency with proportion for categorical variables and the median with interquartile range for continuous variables. Bivariate analyses were conducted to assess an association between each of the factors and the presence of boiled water, using Fisher’s exact test.

**Results:**

Among the 108 households that reported boiling practice, 91 households were able to show the surveyor self-reported boiled water. Thus, the proportion of households with the presence of boiled water was 90.1% (95% confidence interval: 82.5–95.1%). Households with a fixed schedule of boiling were significantly more likely to present boiled water, compared to households without (94.5% vs. 50.0%). Not all household members do not necessarily drink boiled water: approximately a quarter (25.7%) of the participants reported that some household members drink unboiled water.

**Conclusions:**

This study showed that among households that reported boiling drinking water, 90.1% were able to present a container with self-reported boiled water. It suggests that the self-reported measure of boiling practices can be valid in the study villages.

**Supplementary Information:**

The online version contains supplementary material available at 10.1186/s41182-024-00626-z.

## Background

Globally, diarrhea is a leading cause of death among children under 5 years and approximately 525,000 children die each year from diarrhea [1, 2]. Diarrhea can be prevented using safe drinking water, sanitation facilities, and hand washing with soap. For safe drinking water in particular, household water treatment (HWT) is important to reduce the risk of diarrhea [3].

Although HWT is critical, the measurement relies chiefly on self-report in most nationwide surveys and research studies. For example, the Demographic and Health Surveys measure HWT practices with the following two questions; (1) *Do you treat your water in any way to make it safer to drink* and (2) *What do you usually do to the water to make it safer to drink *[4, 5].

Self-report of HWT practices can be biased because people tend to over-report their practices [6]. Some studies report that people are over-reporting their HWT practices. A study in Zambia found that among respondents who reported doing HWT, only 23.1% had household-treated water during the survey [7]. A similar study in Peru found that only 21.3% had household-treated water [8]. In addition, a similar study in Cambodia also found that 73.7% had household-treated water [9]. Thus, the validity of self-reported measurement of HWT practices is of concern.

Lao People’s Democratic Republic (Lao PDR) is a lower-middle-income country located in Southeast Asia. Diarrhea is a common disease and there are challenges related to drinking water in the rural area [10]. Chlorination is not widespread, and the source of drinking water is often contaminated with *Escherichia coli* (*E. coli*): 83.3% of the household population were exposed to *E. coli* in the source of their drinking water [11].

The Lao Social Indicator Survey II (LSIS II) is a household-based nationwide survey conducted by the Lao government in 2017 [11]. According to the LSIS II, in rural areas without roads, 59.8% of households treated their drinking water by boiling, 37.8% did not treat and 4.2% treated it by straining through a cloth. As is done in Demographic and Health Surveys, the HWT practices were measured by self-report in the LSIS II. Therefore, the validity of the measurement is also of concern in the LSIS II.

As far as we know, no study has been conducted to determine the proportion of the presence of boiled water among households that report boiling practices in Lao PDR. The objective of the present study was to determine the proportion of households with the presence of boiled water among households that report boiling practices in a rural area of the Lao PDR.

## Methods

### Study design

This is a descriptive study that described (1) the proportion of households with the presence of boiled water among households that report boiling as HWT and (2) the boiling-related characteristics of households.

### Study site and population

This study was conducted in the four villages in the catchment area of the Salan Health Center and Lako Health Center, in Xepon district of the Savannakhet province, between September and October 2023. Xepon district is located on the Vietnamese border, approximately 600 km to the southeast of the Vientiane capital. According to the Xepon District Health Department, the population of the district in 2023 is 65,000. Most of the people are ethnic minorities, including Tri and Makong people. Xepon was chosen from many rural districts of the Lao PDR because we have established relationship with Xepon District Health Department and Savannakhet Provincial Health Department through the long-term research collaboration.

There are 12 health centers in Xepon District. Of these, Salan Health Center and Lako Health Center were chosen because people there do not buy bottled water and thus, they are expected to boil their drinking water. Four villages exist in the catchment area of the Salan Health Center, whereas eleven villages exist in the catchment area of the Lako Health Center. Two villages were selected from the catchment area of each health center, based on the following two criteria; (1) safely accessible during the rainy season and (2) large enough to meet the sample size requirement (27 or more households). In the area of the Salan Health Center, two villages met the criteria, and those two villages were selected. In the area of the Lako Health Center, three villages met the criteria, and the two closest villages were selected.

From each of the selected villages, 27 households were randomly selected using a household list. The inclusion criterion of households was the households that boil their drinking water.

The exclusion criteria were (1) households that had lived in the village for less than 3 months, (2) households that had not boiled their drinking water in the past 7 days, and (3) households without a member aged 18 or over. Seven households did not meet the selection criterion and no households met the exclusion criteria. In total, 108 households that met the criteria were invited to participate in the study. All the invited households participated in the study.

### Survey and data collection

Surveyors conducted interviews with an adult household member and observations on boiled water through household visits, using a questionnaire (additional file 1) that was developed based on HWT-related studies [7–9, 11]. One day before the household visits, villagers were requested that one adult member in charge of boiling water stay at home on the day of the survey. In the interview, surveyors collected boiling-related information including boiling practices and perception on boiling, and socio-economic and demographic information. In the observation, surveyors observed the presence of self-reported boiling water and facilities and goods related to boiling, including kitchen stoves and kettles.

### Outcome variable

The outcome variable was the presence of boiled water in the household at the time of the survey. To measure the outcome, we asked the following question to the interview respondent “Could you show us boiled water?” and then, observed the water the respondent showed us. We defined the presence of boiled water as the state where a participant is able to show the surveyor a kettle or other container that contains self-reported boiled water.

### Sample size

The sample size was calculated using a confidence interval of ± 10%, a confidence level of 95%, and an estimated proportion of households with boiled water of 50%. The proportion of households that have boiled water was estimated from the results of similar studies [7, 9, 12]. As a result of the calculation, the required number of households was 97. On the assumption that the participation rate of invited households is 90%, we needed to invite 108 households in order to have 97 participating households. The calculation was performed using EZR [13].

### Statistical methods

This study used three statistical approaches. First, a 95% confidence interval was calculated for the proportion of households with the presence of boiled water among households that report boiling practices. Second, descriptive statistics were conducted to summarize the collected information using the frequency with proportion for categorical variables and the median with interquartile range for continuous variables. Third, bivariate analyses were conducted to assess an association between each of the factors and the presence of boiled water, using Fisher’s exact test.

## Results

### Presence of self-reported boiled water

Data were collected from 108 households that reported boiling practice. The data of the seven households were excluded, due to missing values in their questionnaires, leaving the data of 101 households.

Among the 101 households, 91 households were able to show the surveyors self-reported boiled water. Thus, the proportion of households with the presence of boiled water among households that report boiling practices was 90.1% (95% confidence interval: 82.5–95.1%.).

### Socio economic and demographic characteristics of study participants

Most of the participants (82.2%) were males (Table 1). The median age was 39 years. The most common educational attainment was "no formal education"(54.5%), followed by "primary level education" (24.8%). Almost all the participants were a farmer (96.0%). Most of the participants (77.2%) were living with a young child, whereas fewer participants (49.5%) were living with an elderly person.

**Table 1:** Demographic and socio-economic characteristics of participants

Characteristics	Total		Households with boiled water		Households without boiled water		*p* value^a^
	*n* (*n* = 101)	%	*n* (*n* = 91)	%	*n* (*n* = 10)	%	
Gender							
Male	83	82.2	73	80.2	10	100.0	0.202
Female	18	17.8	18	19.8	0	0.0	
Age							
18–29 years	30	29.7	25	27.5	5	50.0	
30–49 years	45	44.6	40	44.0	5	50.0	
50 years and more	26	25.7	26	28.6	0	0.0	
Median (inter-quartile range): years	39 (27–50)		40 (27–50)		31 (24.5–38.8)		
Educational attainment							
No formal education	55	54.5	51	56.0	4	40.0	0.324
Primary school	25	24.8	20	22.0	5	50.0	
Secondary school or higher	21	20.8	20	22.0	1	10.0	
Main source of income							
Farming	97	96.0	87	95.6	10	100.0	1.000
Company officer	4	4.0	4	4.4	0	0.0	
Living with a child under 5 years of age							
Yes	78	77.2	69	75.8	9	90.0	0.448
No	23	22.8	22	24.2	1	10.0	
Living with a person aged 60 years or over							
Yes	50	49.5	44	48.4	6	60.0	0.525
No	51	50.5	47	51.6	4	40.0	
Wealth index							
First (least poor)	32	31.7	30	33.0	4	40.0	0.767
Second	35	34.7	31	34.1	4	40.0	
Third (poorest)	34	33.7	30	33.0	2	40.0	
Floor material							
Wood	58	57.4	51	56.0	7	70.0	
Bamboo	33	32.7	30	33.0	3	30.0	
Cement	10	9.9	10	11.0	0	0.0	
Wall material							
Wood	52	51.5	48	52.7	4	40.0	
Bamboo	45	44.6	39	42.9	6	60.0	
Brick	4	4.0	4	4.4	0	0.0	

^a^Fisher’s exact test

### Boiling practices

**Fig. 1:**
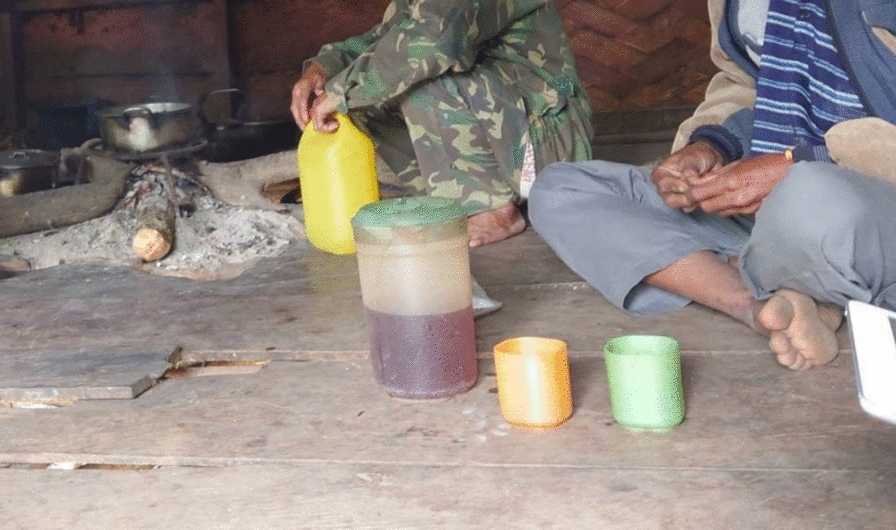
Boiled water in a pitcher

**Fig. 2:**
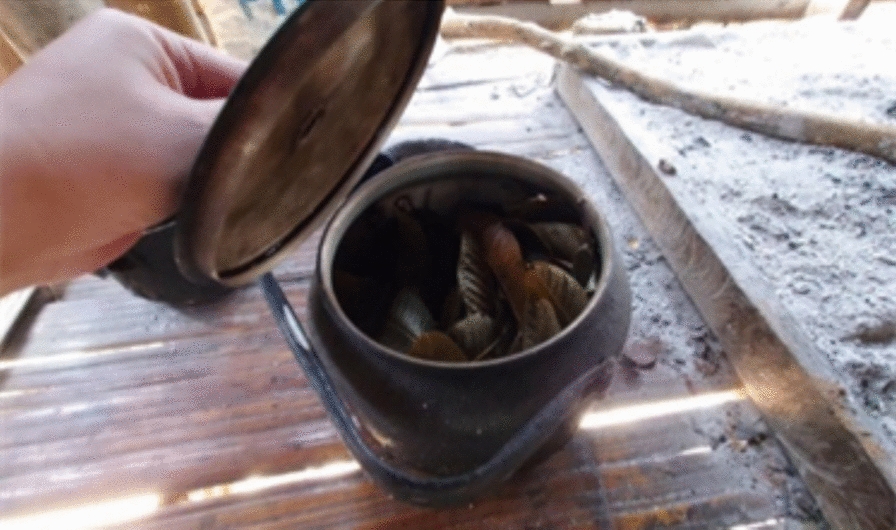
Kettle with tea leaves

Except for two participants, all the participants boil their drinking water using an open cooking stove with wood fuel (Table 2). The most common place for boiling water was outside the living room but inside the house (59.4%), followed by inside the living room (24.8%). A kettle (92.1%) was the most common tool for boiling water. Most of the participants (96.1%) owned only one or two kettles/other boiling tools. The most common type of container for boiled water was kettles (37.6%), followed by pitchers (30.7%) and bottles (29.7%) (Fig. 1). Most of the participants (96.0%) boiled water on the day of the survey or 1 day before the survey. The majority of the participants (90.1%) have a fixed schedule for boiling water: most of the participants (94.1%) boil water every day: the most common time for boiling water is in the morning (84.2%). Almost all participants (95.0%) put something (mostly leaf) in water when boiling water (Fig. 2). The most common reason for putting something was to improve the taste (72.9%), followed by to boil water fast (13.5%).

**Table 2:** Water boiling-related information

Characteristics	Total		Households with boiled water		Households without boiled water		*p* value
	*n* (*n* = 101)	%	*n* (*n* = 91)	%	*n* (*n* = 10)	%	
Presence of boiled water (95%confidence intervals)	(82.5–95.1%)						
How to boil the water							
Open cooking stove	99	98.0	90	98.9	9	90.0	0.189
Electric pot	2	2.0	1	1.1	1	10.0	
Fuel material	**(n = 99)**		**(n = 90)**		**(n = 9)**		
Wood	99	100.0	90	100.0	9	100.0	
Fuel stockpile	**(n = 99)**		**(n = 90)**		**(n = 9)**		
For within a week	36	36.4	32	35.6	4	44.4	0.367
For 1–3 weeks	16	15.2	15	16.7	0	0.0	
For 4 weeks or longer	48	48.5	43	47.8	5	55.6	
Place of boiling							
Outside the living room but inside the house	60	59.4	55	60.4	5	50.0	0.714
Inside the living room	25	24.8	22	24.2	3	30.0	
Outside the house	16	15.8	14	15.4	2	20.0	
Tool of the boiling							
Kettle	93	92.1	85	93.4	8	80.0	0.529
Pot	6	5.9	5	5.5	1	10.0	
Electronic pot	2	2.0	1	1.1	1	10.0	
Number of boiling tools							
One	63	62.4	55	60.4	8	80.0	0.664
Two	34	33.7	32	35.2	2	20.0	
Three	4	4.0	4	4.4	0	0.0	
Type of the container for boiled water							
Kettles	38	37.6	29	31.9	2	20.0	0.109
Picher	31	30.7	25	27.5	5	50.0	
Bottles	30	29.7	36	39.6	2	20.0	
Electric pot	2	2.0	1	1.1	1	10.0	
When was the last time you or other member boiled water?							
Today/yesterday	97	96.0	89	97.8	8	80.0	0.048
2–3 days before	2	2.0	1	1.1	1	10.0	
4 ≥ days before	2	2.0	1	1.1	1	10.0	
Do you have a fixed schedule for boiling water?							
Yes	91	90.1	86	94.5	5	50.0	< .001
No	10	9.9	5	5.5	5	50.0	
Frequency of boiling water in a week							
Everyday	95	94.1	88	96.7	7	70.0	0.012
3 or 4 times a week	5	5.0	3	3.3	2	20.0	
1 or 2 times a week	1	1.0	0	0.0	1	10.0	
Timing of boiling in a day							
In the morning	85	84.2	78	85.7	7	70.0	0.128
In the evening	3	3.0	2	2.2	1	10.0	
Whenever necessary	13	12.9	11	12.1	2	20.0	
Do you put something in water during/after boiling?							
Yes	96	95.0	87	95.6	9	90.0	0.413
No	5	5.0	4	4.4	1	10.0	
What do you put in water?	**(n = 96)**		**(n = 87)**		**(n = 9)**		
Leaf	86	85.1	77	88.5	9	100.0	1.000
Branch	3	3.0	3	3.4	0	0.0	
Root	6	5.9	6	6.9	0	0.0	
Vietnam tea leaf	1	1.0	1	1.1	0	0.0	
Reason for putting things in water	**(n = 96)**		**(n = 87)**		**(n = 9)**		
To taste good	70	72.9	66	75.9	4	44.4	
To boil water fast	13	13.5	11	12.6	2	22.2	
To improve water for health purposes	7	7.3	6	6.9	1	11.1	
To clean water	5	5.2	4	4.6	1	11.1	
I don’t know	1	1.0	0	0.0	1	11.1

### Source of drinking water and preferences

The most common source of drinking water was piped spring (43.5%), followed by groundwater (22.2%) and spring (17.6%) (Table 3). Most of the participants (74.3%) reported that they can get to their water sources within 5 min. However, some participants (15.8%) need over 10 min to reach the water sources.

**Table 3:** Water source, perception and belief

Characteristics	Total		Households with boiled water		Households with no boiled water		*p* value
	*n* (*n* = 101)	%	*n* (*n* = 91)	%	*n* (*n* = 10)	%	
Main source of drinking water (Multiple choices were allowed)	**(*n* = 108)**		**(*n* = 97)**		**(*n* = 11)**		
Piped spring	47	43.5	46	47.4	1	9.1	
Groundwater	24	22.2	20	20.6	4	36.4	
Spring	19	17.6	15	15.5	4	36.4	
Tap water	12	11.1	12	12.4	0	0.0	
Well	6	5.6	4	4.1	2	18.2	
Time required to reach the water source							
Within 5 min	75	74.3	72	79.1	3	30.0	< .001
6–10 min	10	9.9	9	9.9	1	10.0	
Over 10 min	16	15.8	10	11.0	6	60.0	
Is it hard for you to boil water?							
Yes	33	32.7	28	30.8	5	50.0	0.288
No	68	67.3	63	69.2	5	50.0	
Which water tastes better, boiled/non-boiled water?							
Boiled water	96	95.0	89	97.8	7	70.0	< .001
Non-boiled water	5	5.0	2	2.2	3	30.0	
Frequency of drinking boiled water in a day							
Every time	75	74.3	75	82.4	0	0.0	< .001
While at home	24	23.8	16	17.6	8	80.0	
While outside	2	2.0	0	0.0	2	20.0	
Who drinks boiled water in your family?							
All the family members	75	74.3	68	74.7	7	70.0	0.866
Only adult member	8	7.9	7	7.7	1	10.0	
Some members: details are unknown	18	17.8	16	17.6	2	20.0	
Do you think drinking boiled water will prevent diarrhea?							
Yes	84	83.2	77	84.6	7	70.0	0.256
No	6	5.9	5	5.5	1	10.0	
I don’t know	11	10.9	9	9.9	2	20.0	

### Water drinking behavior, perception and belief about boiling

Most of the participants (67.3%) perceived that boiling drinking water is not hard. For most of the participants (95.0%), boiled water taste better than non-boiled water. Although most of the participants (74.3%) drink boiled water every time, approximately a quarter (23.8%) of the participants drink boiled water only when they are at home. In most of the households (74.3%), all the family members drink boiled water. However, in the remaining households (25.7%), not all family members do not necessarily drink boiled water, although no data were available on members who do not drink boiled water. Most of the participants (83.2%) believed that drinking boiled water can prevent diarrhea.

### Results of Fisher’s exact test

There were statistically significant differences between households with boiled water and households without boiled water regarding water boiling-related information; the last time when water was boiled (*p* = 0.048), the presence of a fixed schedule of boiling (*p* < 0.001), and the frequency of boiling water in a week (*p* = 0.012). There were statistically significant differences in the following variables regarding water source, perceptions, and beliefs; time required to reach the water source (*p* < 0.001), preference for drinking water (*p* < 0.001), and frequency of drinking boiled water in a day (*p* < 0.001).

## Discussion

### High presence of boiled water

To be the best of our knowledge, this study is the first study that assessed the gap between self-reported measurement and observational measurement for household boiling water practice in Lao PDR. This study showed that among the households that reported boiling as HWT, 90.1% were able to show water that seems to have been boiled. It suggests that the validity of the self-reported measure of boiling practices is high in the study site. There are at least two possible reasons for the high proportion. First, most of the participants (95.0%) put something (mostly leaves) in their water. In these areas, most people drink tea for drinking water. These areas are located near Vietnam thus the tea-drinking habit might come from Vietnamese culture [14]. The tea-drinking habit may have led to the high proportion of boiled water. Second, most of the participants (83.2%) believed that drinking boiled water can prevent diarrhea, suggesting that knowledge about the importance of boiling is a contributing factor. In fact, a study conducted in Ethiopia reported that knowledge about drinking water is associated with better HWT practices [15, 16].

### Statistical significance of daily scheduled boiling water

Among the factors that were statistically significantly associated with the presence of boiled water, we should emphasize “having a fixed schedule for boiling water”: households that have a fixed schedule for boiling water were approximately two times more likely to have self-reported boiled water, compared to households that do not. Setting up a fixed schedule can be adopted by households without additional resources. Thus, we emphasize the association.

### Dominance of male participants

Most of the participants (82.2%) were male. This may be because of the traditional division of gender roles in the region. A child nutrition study in Savannakhet reported that fathers often enjoyed greater autonomy than mothers [17]. This gender gap may have been a barrier to women's participation. However, the main point of this study on presence of boiled water, there is no difference in results between male and female (male 88.0%, female 100%).

### Place of the boiling water

The present study also found that 24.8% of study participants boil water inside the living room. Boiling in the living room carries several health risks. The smoke produced by boiling can cause respiratory problems [18]. In addition, exposure to boiling water during or immediately after boiling risks serious burns. This risk is especially high in households with small children. Therefore, boiling should be done outside the living room.

### Perception of drinking boiled water

This study also showed that, even though boiled water is available at household, some household members including young children do not drink boiled water. This suggests that the investigation at household-level alone is not enough: water-drinking behavior at individual level should also be investigated for better assessing drinking water-based risk of diarrhea.

## Limitations

There are two major limitations of the study. First, the measurement of the presence of boiled water may be inaccurate, because of no direct observation of the actual boiling process. Therefore, some of the participant-identified boiled water might not have been boiled. The present study used a proxy observation which is used when it is difficult to observe the actual object of a study directly. The proxy observation of boiling practice in the present study is considered to be reliable for the following two reasons. First, a study with rural households in Cambodia reported that the participant-identified boiled water showed significantly lower microbial indicators compared to the participant-identified pre-treatment water [12]. Second, during the household survey, when the surveyors asked study participants to show boiled water, most of the study participants showed brown color water (i.e., tea water). These pieces of information could serve as evidence that the participant-identified water was actually boiled. The second limitation is that, the study area was confined to the purposively selected four villages in the two health center zones. Thus, the applicability of the present study’s findings to a wider area is of concern. However, the present study’s findings can be applicable to a wider area of the Xepon district, because the study participants’ ethnic group (i.e., Tri group) is widely seen in Xepon district [19].

## Conclusion

The present study showed that among households that reported boiling drinking water, 90.1% (95% CI 82.5–95.1%) were able to show a container with water that seems to have been boiled. It suggests that the self-reported measure of boiling practices is valid in the study villages. A further study in randomly selected villages from a wider area is recommended to confirm the findings of the present study.

## Supplementary Information

Additional file 1.

## Data Availability

The data sets generated or analyzed in the current study are available from the corresponding author upon a reasonable request.

## References

[1] United Nations Inter-agency Group for Child Mortality Estimation (UN IGME). Levels & trends in child mortality: report 2021 developed by the United Nations Inter-agency Group for Child Mortality Estimation. New York: United Nations Children’s Fund; 2021.

[2] World Health Organization. Diarrhoeal disease. https://www.who.int/news-room/fact-sheets/detail/diarrhoeal-disease. Accessed 1 Aug 2024.

[3] Clasen T, Boisson S, Routray P, Torondel B, Bell M, Cumming O, Ensink J, Freeman M, Jenkins M, Odagiri M, Ray S, Sinha A, Suar M, Schmidt WP. Effectiveness of a rural sanitation programme on diarrhoea, soil-transmitted helminth infection, and child malnutrition in Odisha, India: a cluster-randomised trial. Lancet Glob Health. 2014;2(11):e645–53. 25442689 10.1016/S2214-109X(14)70307-9

[4] DHS program. Who we are. https://dhsprogram.com/Who-We-Are/About-Us.cfm. Accessed 1 Aug 2024.

[5] WHO, UNICEF. Core questions on drinking water and sanitation for household surveys. Geneva: World Health Organization; 2006.

[6] Chidziwisano K, Tilley E, Morse T. Self-reported versus observed measures: validation of child caregiver food hygiene practices in rural Malawi. Int J Environ Res Public Health. 2020;17(12):4498. 32585833 10.3390/ijerph17124498 PMC7344643

[7] Rosa G, Kelly P, Clasen T. Consistency of use and effectiveness of household water treatment practices among urban and rural populations claiming to treat their drinking water at home: a case study in Zambia. Am J Trop Med Hyg. 2016;94(2):445–55. 26572868 10.4269/ajtmh.15-0563 PMC4751932

[8] Rosa G, Huaylinos ML, Gil A, Lanata C, Clasen T. Assessing the consistency and microbiological effectiveness of household water treatment practices by urban and rural populations claiming to treat their water at home: a case study in Peru. PLoS ONE. 2014;9(12): e114997. 25522371 10.1371/journal.pone.0114997 PMC4270781

[9] Benwic A, Kim E, Khema C, Phanna C, Sophary P, Cantwell RE. Factors associated with post-treatment *E. coli* contamination in households practising water treatment: a study of rural Cambodia. Int J Environ Health Res. 2018;28(2):178–91. 29575938 10.1080/09603123.2018.1453055

[10] GBD 2016 Diarrhoeal Disease Collaborators. Estimates of global, regional, and national morbidity, mortality, and aetiologies of diarrhoeal diseases: a systematic analysis for the global burden of disease study 2015. Lancet Infect Dis. 2017;17:909–48. 28579426 10.1016/S1473-3099(17)30276-1 PMC5589208

[11] Lao Statistics Bureau. Lao Social Indicator Survey II (LSIS II). https://www.unicef.org/laos/media/306/file/LSIS2017ENG.pdf Accessed 1 Aug 2024.

[12] Brown J, Sobsey MD. Boiling as household water treatment in Cambodia: a longitudinal study of boiling practice and microbiological effectiveness. Am J Trop Med Hyg. 2012;87(3):394–8. 10.4269/ajtmh.2012.11-0715. 22826487 10.4269/ajtmh.2012.11-0715 PMC3435338

[13] Kanda Y. Investigation of the freely available easy-to-use software ‘EZR’ for medical statistics. Bone Marrow Transpl. 2013;48(3):452–8. 10.1038/bmt.2012.244 PMC3590441 23208313

[14] Robert W. The deep roots of Vietnamese tea: culture, production and prospects for development. Independent Study Project (ISP) Collection. 2011:1159. https://digitalcollections.sit.edu/isp_collection/1159/.

[15] Bitew BD, Gete YK, Biks GA, Adafrie TT. Knowledge, attitude, and practice of mothers/caregivers on household water treatment methods in northwest Ethiopia: a community-based cross-sectional study. Am J Trop Med Hyg. 2017;97(3):914–22. 10.4269/ajtmh.16-0860. 28722624 10.4269/ajtmh.16-0860 PMC5590582

[16] Sisay W, Tsadik D, Debela BG, Ali Ewune H, Hareru HE. Determinants of household-level water treatment practices in southern Ethiopia. Environ Health Insights. 2022. 10.1177/11786302221109399. 35782318 10.1177/11786302221109399 PMC9243476

[17] Boulom S, Bon DM, Essink D, Kounnavong S, Broerse JEW. Understanding discrepancies in nutritional outcomes among under-fives in Laos: a mixed-methods study using the positive deviance approach. Food Nutr Bull. 2022;43(3):303–22. 35506170 10.1177/03795721221096187 PMC9403390

[18] Mengersen K, Morawska L, Wang H, Murphy N, Tayphasavanh F, Darasavong K, Holmes N. The effect of housing characteristics and occupant activities on the respiratory health of women and children in Lao PDR. Sci Total Environ. 2011;409(8):1378–84. 10.1016/j.scitotenv.2011.01.016. 21300397 10.1016/j.scitotenv.2011.01.016

[19] Inthavong N, Nonaka D, Kounnavong S, Iwagami M, Phommala S, Kobayashi J, Hongvanthong B, Pongvongsa T, Brey PT, Kano S. Individual and household factors associated with incidences of village malaria in Xepon district, Savannakhet province, Lao PDR. Trop Med Health. 2017;7(45):36. 10.1186/s41182-017-0077-2. 10.1186/s41182-017-0077-2 PMC5678595 29151802

