# Supplementary material for: Do they really boil their drinking water? a descriptive study in a rural district of the Lao people’s democratic republic
Source: Trop Med Health. 2024 Sep 18;52:60. doi: 10.1186/s41182-024-00626-z (PMC11409578; doi:10.1186/s41182-024-00626-z)
Supplement: Supplementary file 1 — Additional file 1. [file 41182_2024_626_MOESM1_ESM.docx]

**Additional file 1 Questionnaire**

**Section 1: Interview**

|  | Question | Answer |
| --- | --- | --- |
|  | How old are you? |  |
|  | What is your gender? | 1) Male 2) Female  3) Others (please specify)  _____________ |
|  | What is your occupation? | 1) Farming  2) Livestock rearing  3) Forestry  4) Handicraft  5) Others (please specify)  ___________ |
|  | Are there any persons aged 5 or younger in this house? | 1) Yes 2) No |
|  | Are there any persons aged 60 or over in this house? | 1) Yes 2) No |
|  | What is the highest level of education you have completed? | 1) No formal education  2) Primary school  3) Secondary school  4) Technical school  5) Vocational school  5) Others (please specify)  _________ |
|  | Does your household have:  A television?  A radio?  A watch?  A motorcycle or scooter?  A car?  A boat?  A Tak Tak?  A bicycle?  Livestock?  ⇀If yes   \| 10-1. Please choose the livestock that your household has.  Multiple choices are allowed \| \| --- \| |  |
|  |  | 1) Yes 2) No |
|  |  | 1) Yes 2) No |
|  |  | 1) Yes 2) No |
|  |  | 1) Yes 2) No |
|  |  | 1) Yes 2) No |
|  |  | 1) Yes 2) No |
|  |  | 1) Yes 2) No |
|  |  | 1) Yes 2) No |
|  |  | 1) Yes 2) No |
|  |  | \| 1) Chicken \| \| --- \| \| 2) Duck \| \| 3) Pig \| \| 4) Goat \| \| 5) Cow \| \| 6) Water buffalo \| |
|  | What is your main source of drinking water?  Multiple choices are allowed. | 1) Groundwater  2) Well  3) Small river  4) Spring with pipe  5) Rainwater  6) Others (please specify)  _________ |
|  | How long does it take to get to the water source from your house?  If there are more than two water sources, answer the more distant one. | 1) Within 5 minutes  2) 6 to 10 minutes  3) Over 10 minutes |
|  | When was the last time you or other members of your household boiled water? | 1) Today or yesterday  2) 2 to 3 days before  3) 4 to 5 days before  4) 6 to 7 days before  5) Forget  6) Others (please specify)  __________ |
|  | Please show me the latest boiled water.  Is there some water? Surveyors check if there is some water and write the observation result. | Observation  1) Yes 2) No |
|  | ⇀ If there is boiled water in the house   \| 12-1. Why do you boil your drinking water? \| \| --- \|   ⇀ If there is no boiled water in the house   \| 12-2. Why is there no boiled water? \| \| --- \| | \|  \| \| --- \|  \|  \| \| --- \| |
|  | Do you put something in the water during or after boiling? (For example, leaf, bark, or root of some plant),  　⇀If yes   \| 13-1) What do you put in the water? \| \| --- \|  \| 13-2) Why do you put it in? \| \| --- \| | 1) Yes  2) No   \| 1) Leaf  2) Branch  3) Others (please specify)  _____________ \| \| --- \|  \| 1) To tastes good  2) To distinguish the boiled water from non-boiled water  3) To clean the water  4) To boil the water early  5) Others (please specify)  _____________ \| \| --- \| |
|  | Do you decide when to boil the water?  ⇀If yes,   \| When do you usually boil the water in a day? \| \| --- \| | 1) Yes  2) No   \| 1) In the morning  2) In the afternoon  3) In the evening  4) Others (please specify)  _________ \| \| --- \| |
|  | Please tell me all the people in charge of boiling water in the household. |  |
|  | How often does your household boil drinking water in a week? | 1) Everyday  2) 6 or 5 times a week  3) 4 or 3 times a week  4) 2 or 1 times a week  5) Others (please specify)  _________ |
|  | Is it hard for you to boil water? | 1) Yes  2) No |
|  | When do you drink boiled water in a day? | 1) Every time  2) While at home  3) While outside  4) Others (please specify)  ________ |
|  | Please tell me all the people who drink boiled water in the household. |  |
|  | Which water tastes better, boiled water or non-boiled water?   \| 27-1. How tasty? \| \| --- \| | 1) Boiled water  2) Non-boiled water   \|  \| \| --- \| |
|  | Do you think drinking boiled water will prevent diarrhea? | 1) Yes  2) No  3) I don’t know  4) Others (please specify)  ________ |

**Section 2: Observation**

|  | Question | Observation results |
| --- | --- | --- |
|  | What is the floor made of? | 1) Wood  2) Bamboo  3) Others (please specify)  ______ |
|  | What is the wall made of? | 1) Wood  2) Bamboo  3) Brick  4) Others (please specify)  _______ |
|  | Could you show us the kitchen?  If yes, surveyors check the result of the observation | 1) Yes 2) No  Observation  Type of cooking stove  1) Open  2) Closed (see Fig. 1)  3) Others (please specify)  ________  Fig.1) Closed cooking stove  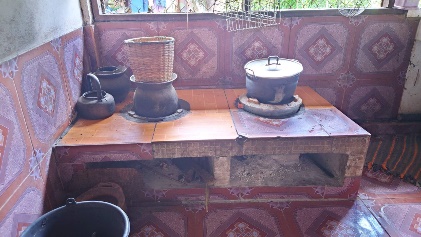  Place of boiling  1) Inside the living room  2) Outside the living room but inside the house  3) Outside the house  4) Others (please specify) _________ |
|  | What is the fuel for boiling water? | 1) Wood  2) Charcoal  3) Others (please specify)  _________ |
|  | How much fuel do you save? | 1) for within a week  2) for one to three weeks  3) four weeks or longer  4) Others (please specify)  _________ |
|  | Which tool do you use kettle or pot during boiling? | 1) Kettle  2) Pot  3) Others (please specify)  _____________ |
|  | How many kettles and pots are there? |  |
|  | What is the container for boiled water in your household? | 1) Picher  2) Bottles  3) Kettles  4) Others (please specify)  __________ |
